# Waist circumference mediates the association between rs1260326 in *GCKR* gene and the odds of lean NAFLD

**DOI:** 10.1038/s41598-023-33753-4

**Published:** 2023-04-20

**Authors:** Na Wu, Jie Li, Jing Zhang, Fan Yuan, Ning Yu, Fengwei Zhang, Dong Li, Jianying Wang, Lei Zhang, Yi Shi, Guang He, Guang Ji, Baocheng Liu

**Affiliations:** 1 Shanghai Innovation Center of Traditional Chinese Medicine Health Service, School of Public Health, Shanghai University of Traditional Chinese Medicine, Shanghai, 201203 China; 2 Bio-X Institutes, Key Laboratory for the Genetics of Developmental and Neuropsychiatric Disorders, Shanghai Jiao Tong University, Shanghai, 200030 China; 3 Institute of Digestive Diseases, Longhua Hospital, Shanghai University of Traditional Chinese Medicine, Shanghai, 200032 China; 4 Zhangjiang Community Health Service Center of Pudong New District, Shanghai, 201210 China; 5 Department of Biochemistry and Molecular Biology, Capital Medical University, Beijing, 100069 China

**Keywords:** Cardiovascular diseases, Endocrine system and metabolic diseases, Genetic association study

## Abstract

While non-alcoholic fatty liver disease (NAFLD) has been widely studied, the pathophysiology of lean NAFLD, the critical NAFLD subgroup, remains elusive. This study aimed to clarify the association between polymorphisms of *GCKR*, waist circumference, and the odds of lean NAFLD in the elderly Chinese Han population who live in the Zhangjiang community center of Shanghai, China. Three single nucleotide polymorphisms (SNPs), including rs1260326, rs780093, and rs780094, were genotyped in MassARRAY Analyzer. The association between SNPs with waist circumference in five genetic models was analyzed and rechecked by the logistic regression analysis. Mediation models were established to evaluate whether the waist circumstance can mediate the association between SNPs and lean NAFLD. In this study, the frequency of the C allele of rs1260326, rs780093, and rs780094 was significantly lower in lean NAFLD individuals than in lean non-NAFLD ones. The association between rs1260326 in *GCKR* and the odds of lean NAFLD was mediated via waist circumference after adjusting gender and age in the elderly Chinese Han population (β = 1.196, R^2^ = 0.043, p = 0.020). For the first time, this study examined the mediating effect of waist circumference on the association between rs1260326 in *GCKR* and the odds of lean NAFLD (β = 0.0515, 95% CI 0.0107–0.0900, p = 0.004). It may contribute to illustrating the pathogenesis of lean NAFLD and indicate that waist circumference management might improve lean NAFLD control.

## Introduction

Non-alcoholic fatty liver disease (NAFLD) is a spectrum progressing from simple steatosis to nonalcoholic steatohepatitis (NASH), fibrosis, cirrhosis, and hepatocellular carcinoma (HCC)^1^, and it has become the predominant cause of chronic liver disease in the world. NAFLD also links tightly to obesity, type II diabetes (T2D), and cardiovascular diseases, which have given rise to an enormous burden on society^2^.

It is worth noting that not all NAFLD patients are overweight or obese, the lean individuals also account for a significant proportion (10–20%) of NAFLD cases in the world^3^. And lean NAFLD might probably progress to NASH and fibrosis^4^, although these patients usually presented fewer obesity-related conditions. Both genetic background and abnormal glucose and lipid, e.g., dyslipidemia and altered glucose and lipid turnover, are possibly involved in the pathological mechanism of lean NAFLD^5^. Besides being used to screen abdominal fat distribution and obesity^6^, waist circumference positively correlated with the risks of lean NAFLD^7^. The distinct role of waist circumference on lean NAFLD has caught wide attention. However, despite the functions of various genetic predispositions in the pathogenesis of NAFLD in obese and lean patients, the relevant research has mainly been studied in obese individuals^8,9^; effects of genetic variation in glucose or lipid-related genes on lean NAFLD-related traits have rarely been studied.

Accumulating evidence indicated that genetic variants have contributed to the occurrence and development of NAFLD^10,11^. Genome-wide association studies (GWASs) have shown several single nucleotide polymorphisms (SNPs), e.g., rs738409-G in patatin‐like phospholipase domain containing 3 (*PNPLA3*)^11^, rs58542926-T in transmembrane 6 superfamily member 2 (*TM6SF2*)^12^, rs13107325-T in solute carrier family 39 member 8 (*SLC39A8*)^13^, rs6834314-A in hydroxysteroid 17-beta dehydrogenase 13 (*HSD17B13*)^14^ and rs1260326-T in glucokinase regulator (*GCKR*)^15,16^ associated with NAFLD-related comorbidities. Amongst the SNPs identified in *GCKR*, e.g., rs780093 and rs780094 were also identified as potential risk loci in metabolic syndrome^17^. Additionally, *GCKR* is involved in glucose and lipid homeostasis by regulating glucokinase, a rate-limiting enzyme in glycolysis^18^. Although the same genetic variants may drive most lean NAFLD individuals as overweight and obese, genetic variation might be influenced by diverse races and regions; the distinct role of SNPs of *GCKR* in lean NAFLD will be explored in the present study.

Considering all these clues, elderly adults are more susceptible to several NAFLD^19^. The prevalence increases rapidly, especially in China^20^; we aimed to clarify the association between three common polymorphisms of *GCKR* (rs1260326, rs780093, and rs780094), waist circumference, and the odds of lean NAFLD in the elderly Chinese Han population. It will provide increasing evidence to support the role of genetic variation in the odds of lean NAFLD occurrence and development.

## Results

### Demographics of study participants

**Figure 1:**
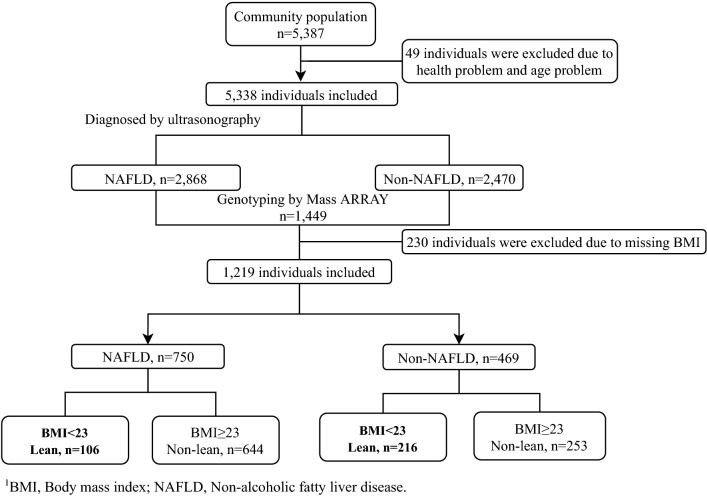
Study flowchart. *BMI* Body mass index, *NAFLD* non-alcoholic fatty liver disease.

In total, 5, 338 potential subjects were included after an initial screening; 2, 868 NAFLD and 2, 470 non-NAFLD participants were distinguished by ultrasonography; 1219 participants (NAFLD, n = 750; non-NAFLD, n = 469) were included for the genotyping analysis; participants were categorized into four groups: lean NAFLD (BMI < 23 kg/m^2^, n = 106), non-lean NAFLD (BMI ≥ 23 kg/m^2^, n = 644), lean non-NAFLD (BMI < 23 kg/m^2^, n = 216) and non-lean non-NAFLD (BMI ≥ 23 kg/m^2^, n = 253) (Fig. 1).

The general characteristics of the lean participants are shown in Table 1. The mean ages of lean NAFLD (n = 106) and lean non-NAFLD (n = 216) were 72.5 and 73.5 years old, respectively. Females and males constitute 64.2 and 35.8 percent of lean NAFLD individuals and 58.8 and 41.2 percent of lean non-NAFLD individuals, respectively. Weight, BMI, waist circumference, hip circumference, waist-to-hip ratio (WHR), ALT, fasting glucose, TC, LDL, and TG were significantly higher in lean NAFLD individuals than in lean non-NAFLD individuals (p < 0.001, p < 0.001, p < 0.001, p < 0.001, p < 0.001, p = 0.014, p = 0.001, p = 0.008, p = 0.008 and p < 0.001, respectively). In contrast, lean NAFLD individuals had decreased levels of HDL (p < 0.001). In contrast, there were no significant differences in blood pressure, AST, and the percentage of hypertension, T2D, and hyperlipidemia between lean NAFLD and lean non-NAFLD individuals.

**Table 1:** Clinical characteristics of lean NAFLD and lean non-NAFLD individuals.

	Lean NAFLD	Lean non-NAFLD	P
	Mean ± SD	Mean ± SD	
N	106	216	
Age (year)	72.54 ± 6.05	73.54 ± 6.15	0.169
Gender (%)			
Female	64.20%	58.80%	0.357
Male	35.80%	41.20%	
Height (cm)	159.13 ± 8.24	158.60 ± 8.35	0.588
Weight (kg)	55.42 ± 6.25	51.92 ± 6.98	< 0.001
BMI (kg/m^2^)	21.83 ± 0.96	20.60 ± 1.78	< 0.001
Waist circumference (cm)	79.94 ± 6.26	75.63 ± 6.92	< 0.001
Hip circumference (cm)	90.43 ± 3.91	88.37 ± 4.85	< 0.001
WHR	0.88 ± 0.06	0.85 ± 0.08	< 0.001
SBP (mmHg)	142.04 ± 20.43	138.04 ± 22.45	0.124
DBP (mmHg)	81.08 ± 12.32	78.83 ± 11.92	0.119
ALT (U/L)	22.44 ± 9.254	19.73 ± 9.28	0.014
AST (U/L)	23.88 ± 10.44	23.53 ± 8.58	0.750
Fasting glucose (mmol/L)	6.70 ± 2.20	5.89 ± 1.66	0.001
TC (mmol/L)	5.29 ± 0.91	4.99 ± 1.01	0.008
HDL (mmol/L)	1.16 ± 0.20	1.33 ± 0.30	< 0.001
LDL (mmol/L)	3.30 ± 0.85	3.05 ± 0.89	0.018
TG (mmol/L)	1.92 ± 1.20	1.18 ± 0.69	< 0.001
Hypertension (%)	55.70	44.00	0.050
T2D (%)	20.80	12.0	0.056
Hyperlipidemia (%)	10.40	6.50	0.221

Variables are presented as mean ± SD. P values are based on independent sample t- test.

*ALT* alanine aminotransferase, *AST* aspartate aminotransferase, *BMI* body mass index, *DBP* diastolic blood pressure, *HDL* high density lipoprotein, *LDL* low density lipoprotein, *NAFLD* nonalcoholic fatty liver disease, *SBP* systolic blood pressure, *TC* total cholesterol, *TG* triglyceride, *T2D* type 2 diabetes, *WHR* waist to hip ratio.

### Genetic association between SNPs and lean NAFLD

Three tested SNPs (rs1260326, rs780093, and rs780094) in *GCKR* shown in Table 2 met Hardy–Weinberg equilibrium (p > 0.05), and the allele and genotype distributions of three SNPs in *GCKR* are shown in Table 3. The frequency of the C allele of rs1260326, rs780093, and rs780094 in *GCKR* was significantly lower in lean NAFLD compared with lean non-NAFLD individuals (OR = 0.700, 95% CI 0.499–0.981, p = 0.038; OR = 0.685, 95% CI 0.488–0.962, p = 0.028; OR = 0.698, 95% CI 0.497–0.980, p = 0.037). The genotypic frequency of these three SNPs on the *GCKR* gene significantly differed between lean NAFLD and lean non-NAFLD individuals (p < 0.05).

**Table 2:** The three SNPs in the *GCKR* gene analyzed in this study.

SNP ID	Chromosome	Function	Allele
rs1260326	Chr2: 27508073	Missense variant	T/C
rs780093	Chr2:27519736	Intron_variant	T/C
rs780094	Chr2:27518370	Intron_variant	T/C

*Chr* chromosome, *GCKR* glucokinase regulator, *SNP* single nucleotide polymorphisms.

**Table 3:** *GCKR* allele and genotype distribution in lean individuals.

SNP	Allele frequency		χ^2^	P	FDR adjusted	OR (95% CI)	Genotype frequency			χ^2^	P	FDR adjusted	HWE
rs1260326	C	T	4.283	0.038	0.162	0.700 (0.499–0.981)	C/C	C/T	T/T	8.392	0.015	0.143	
Lean NALFD	79 (0.376)	131 (0.623)					12 (0.114)	55 (0.523)	38 (0.361)				0.493
Lean non-NAFLD	198 (0.462)	230 (0.537)					54 (0.252)	90 (0.42)	70 (0.327)				0.078
rs780093	C	T	4.787	0.028	0.378	0.685 (0.488–0.962)	C/C	T/T	C/T	6.294	0.042	0.449	
Lean NALFD	80 (0.388)	126 (0.611)					13 (0.126)	36 (0.349)	54 (0.524)				0.575
Lean non-NAFLD	202 (0.48)	218 (0.519)					52 (0.247)	60 (0.285)	98 (0.466)				0.638
rs780094	C	T	4.313	0.037	0.378	0.698 (0.497–0.980)	C/C	T/T	C/T	6.204	0.044	0.449	
Lean NALFD	80 (0.388)	126 (0.611)					13 (0.126)	36 (0.349)	54 (0.524)				0.575
Lean non-NAFLD	200 (0.476)	220 (0.523)					52 (0.247)	62 (0.295)	96 (0.457)				0.479

*FDR* false discovery rate, *GCKR* glucokinase regulator, *HWE* Hardy–Weinberg equilibrium, *OR* odds ratio, *SNP* single nucleotide polymorphisms.

### Association of SNPs in *GCKR* with waist circumference in lean NAFLD individuals

The association using five genetic models is presented in Table 4. *GCKR* polymorphism rs1260326 was significantly associated with waist circumference under the dominant and over-dominant models. The TT genotype of *GCKR* rs1260326 polymorphism was statistically related to smaller waist circumference (p = 0.023). We also used rs1260326 as a logistic regression predictor to examine the associations with waist circumference. And rs1260326 genotype was still significantly associated with waist circumference (β = 1.196, R^2^ = 0.043, p = 0.020) after adjusting gender and age (Table 5).

**Table 4:** Association between *GCKR* SNPs and waist circumference.

Association	Genotype	N	95% CI	p-value
rs1260326-waist circumference	Codominant			
	T/T	105		0.054
	T/C	142	0.411 to 3.903	
	C/C	65	− 0.819 to 3.462	
	Dominant			
	T/T	105		0.023
	T/C-C/C	207	0.271 to 3.519	
	Recessive			
	T/T-T/C	247		0.933
	C/C	65	− 1.824 to 1.987	
	Over-dominant			
	T/T-C/C	170		0.037
	T/C	142	0.109 to 3.195	
	log-Additive			
	0,1,2		− 0.235 to 1.887	0.128
rs780093-waist circumference	Codominant			
	T/T	94		0.216
	T/C	149	− 0.332 to 3.263	
	C/C	64	− 0.606 to 3.818	
	Dominant			
	T/T	94		0.081
	T/C-C/C	213	− 0.1795 to 3.195	
	Recessive			
	T/T-T/C	243		0.472
	C/C	64	− 1.216 to 2.629	
	Over-dominant			
	T/T-C/C	158		0.307
	T/C	149	− 0.746 to 2.376	
	log-Additive			
	0,1,2		− 0.230 to 1.961	0.123
rs780094-waist circumference	Codominant			
	T/T	96		0.183
	T/C	147	− 0.238 to 3.342	
	C/C	64	− 0.555 to 3.847	
	Dominant			
	T/T	96		0.066
	T/C-C/C	211	− 0.096 to 3.257	
	Recessive			
	T/T-T/C	243		0.472
	C/C	64	− 1.216 to 2.629	
	Over-dominant			
	T/T-C/C	160		0.263
	T/C	147	− 0.668 to 2.455	
	log-Additive			
	0,1,2		− 0.196 to 1.984	0.109

*CI* confidence interval, *GCKR* glucokinase regulator, *SNP* single nucleotide polymorphisms.

**Table 5:** Logistic regression analysis on the impacts of *GCKR* rs1260326 on waist circumference after the adjustment of gender and age.

Phenotype	SNP	Statistics of regression analyses			
		β	R^2^	t	p
Waist circumference	rs1260326	1.916	0.043	2.343	0.020

The T/T genotype was used as reference.

*GCKR* glucokinase regulator, *SNP* single nucleotide polymorphism.

### Mediation effect of waist circumference on the association between rs1260326 and the odds of lean NAFLD

**Figure 2:**
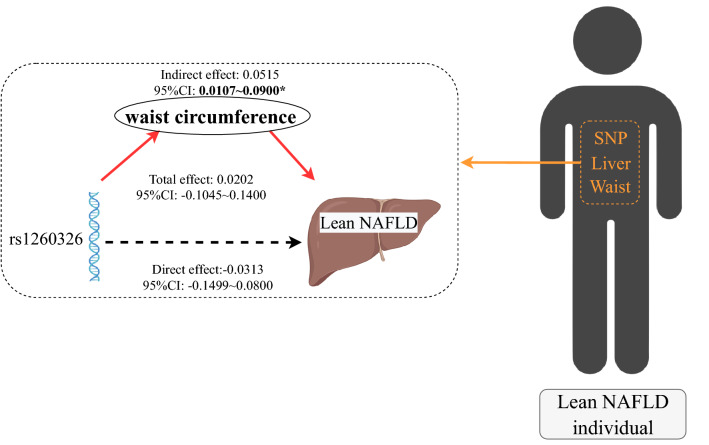
Mediation of waist circumference on the association between rs1260326 and lean NAFLD. *NAFLD* non-alcoholic fatty liver disease. In this study, rs1260326-C had no significant direct effect on lean NAFLD (95% CI − 0.1499 to 0.0800), rs1260326-C had a significant indirect effect on lean NAFLD incidence via waist circumference (95% CI 0.0107–0.0900). Zero was not included in 95% confidence intervals representing statistical significance (p = 0.004).

The mediation analysis indicated that rs1260326-C had no significant direct effect on lean NAFLD (β = − 0.0313, 95% CI − 0.1499 to 0.0800), while rs1260326-C had a significant indirect effect on lean NAFLD incidence via waist circumference (β = 0.0515, 95% CI 0.0107–0.0900, p = 0.004) (Fig. 2).

## Discussion

For the first time, this study examined the mediating effect of waist circumference on the association between rs1260326-C in the *GCKR* gene and the odds of lean NAFLD in the elderly Chinese Han population. And three SNPs, i.e., rs1260326, rs780093, and rs780094, were all associated with the odds of lean NAFLD in the elderly Chinese Han population. In addition, the frequency of the C allele of rs1260326, rs780093, and rs780094 was significantly lower in lean NAFLD individuals than in lean non-NAFLD ones.

The pathophysiological mechanism underlying the occurrence and development of lean NAFLD is still elusive. Waist circumference can be a better alternative for clinically evaluating abdominal obesity and predicting the risk of metabolic diseases^21^ as BMI fails to discriminate subcutaneous fat from visceral fat accurately^22^. Furthermore, WHR also presents the degree of body fat distribution^23^, associated with cardiovascular diseases^24^. While Neeland et al.^25^ showed that the association between visceral fat and WHR was much weaker when compared with waist circumference. Thus, waist circumference was recommended for assessing the odds of obesity other than the single measurement of BMI or WHR^21–25^. Based on the above evidence, we checked the potential role of waist circumference in the odds of lean NAFLD. And the study showed a significant decrease in waist circumference in lean NAFLD than in lean non-NAFLD individuals. It may imply that waist circumference reflects the lipid metabolism dysfunction in lean NAFLD patients. The finding supported this implication, which showed that waist circumference was highly related to NAFLD or specific lean NAFLD^7,26^.

It is well known that NAFLD and its related traits, such as waist circumference, are determined by genetic variants combined with environmental factors. Tong et al.^27,28^ found that rs1044250-C in angiopoietin-like protein 4 (*ANGPTL4*) and rs237025-A in small ubiquitin-like modifier 4 (*SUMO4*) were associated with increased waist circumference in patients with metabolic syndrome. Duicu et al.^29^ confirmed that rs9939609-A in fat mass and obesity-related (*FTO*) gene was a risk factor for waist circumference in obesity. However, there were few studies on waist circumference-related single nucleotide polymorphisms in lean NAFLD. The association between rs738409 GG genotype in *PNPLA3* and the risk of lean NAFLD was reported in citizens of Hongkong, China^30^, and rs58542926-T in *TM6SF2* was associated with TG, not waist circumference in lean NAFLD individuals^30^. Of note, we first discovered that rs1260326-C in the *GCKR* gene was linked to waist circumference in the elderly Chinese Han population with lean NAFLD. This connection may be attributed to the disrupted glucose and lipid metabolism regulated by *GCKR*^31^, leading to abnormal waist circumference.

Interestingly, there was a significant relation between rs1260326-T and rs780094-T polymorphisms of *GCKR* and the increased risk of NAFLD^32^. Hernaez et al.^33^ showed a correlation between patients with simple steatosis with higher ALT and rs780094-T. Tan et al.^34^ demonstrated that NAFLD patients with rs1260326 and rs780094 allele T were prone to progress to NASH with significant fibrosis, which would be developed from lean NAFLD^4^. In addition, Yuan et al.^16^ explored the mediation effect of TG on the association between rs1260326-T in *GCKR* and the risk of NAFLD in the elderly Chinese Han population. Yet, in our study, the association between rs1260326-C and the odds of lean NAFLD was mediated by waist circumference. All these findings emphasize the importance of *GCKR* not only in NAFLD but in the specific lean NAFLD.

The limitation of this study is that the association between *GCKR* polymorphisms and the odds of lean NAFLD was only observed in the elderly Chinese Han population. Since multiple factors affect gene variation, more research in different ethnic groups and regions with larger sample sizes is needed to verify the current result. Although the mediating effect of waist circumference on the association between rs1260326-C in *GCKR* and the odds of lean NAFLD in the elderly Chinese Han population was explored in this study, we still cannot ignore that the temporal link between the outcome and the exposure cannot be determined because both are examined at the same time in a cross-sectional study. More data are needed from the longitudinal study to verify the mediation association. Additionally, the information of health condition, e.g., hypertension, T2D, and hyperlipidemia were determined according to the patient’s self-report; this may cause errors in record, recall, and social desirability bias.

In conclusion, the mediating effect of waist circumference on the association between rs1260326-C in *GCKR* and the odds of lean NAFLD in the elderly Chinese Han population was explored for the first time. This finding may contribute to illustrating the pathogenesis and progression of lean NAFLD and indicate that waist circumference management might improve lean NAFLD control.

## Materials and methods

### Subjects

This study was a cross-sectional investigation conducted in the Zhangjiang community center Shanghai, China. A total of 5387 residents (aged ≥ 60 years) were recruited in 2017. The study was approved by the Ethics Committee of the Shanghai University of Traditional Chinese Medicine. All participants provided informed written consent prior to the study. The study followed the declaration of Helsinki.

The inclusion criteria: residents in Shanghai, complete data measurements. The exclusion criteria: participants with mental disorders, malignant tumors, or incomplete recorded information. After an initial screening, 5,338 potential subjects were included, and 49 individuals were excluded due to health problems and age problems (age < 60 years). Then 1,449 participants were randomly chosen for the genotyping analysis. After further screening, 230 participants who lacked BMI data, abused alcohol (< 140 g/week in males and < 70 g/week in females), were carriers of hepatitis B or C, or had a history of drug-induced liver disease or autoimmune liver disease were excluded, and 1219 participants (NAFLD, n = 750; non-NAFLD, n = 469) were included for the final analysis. According to the classification in adult Asian populations^35^, lean NAFLD in this study was defined by body mass index (BMI) < 23 kg/m^2^. Finally, participants were categorized into four groups: lean NAFLD (BMI < 23 kg/m^2^, n = 106), non-lean NAFLD (BMI ≥ 23 kg/m^2^, n = 644), lean non-NAFLD (BMI < 23 kg/m^2^, n = 216) and non-lean non-NAFLD (BMI ≥ 23 kg/m^2^, n = 253) (Fig. 1).

### Measurement

The NAFLD was diagnosed^36^ and evaluated by the color ultrasound system. Collection of information such as age, gender, alcohol consumption, smoking, and medical history (e.g., hypertension, osteoporosis and cerebral infarction) was collected by questionnaire (Supplementary Table S1). Hypertension, T2D, and hyperlipidemia were determined according to the patient’s self-report, which was acquired from the doctors. Alcohol consumption and smoking were divided into two categories (never and always use) by the following questions, respectively: “Have you ever used tobacco?” and “Have you ever used alcohol?” Body mass index (BMI) was calculated as weight (kg) divided by height squared (m^2^). And participants were categorized into two groups: lean (BMI < 23 kg/m^2^) and non-lean (BMI ≥ 23 kg/m^2^). And lean NAFLD in the present study was defined by BMI < 23 kg/m^2^ according to the classification in adult Asian populations^37^. Waist and hip circumference were reliably measured using a non-stretch tape by the trained professional; waist circumference was measured midway between the lowest rib and the top of the iliac crest at the end of gentle expiration, hip circumference was measured over the great trochanters; circumferences were measured over the naked skin and noted to the nearest 0.1 cm. Blood pressure was measured by electronic sphygmomanometers (Biospace, Cheonan, South Korea). Fasting glucose, alanine transaminase (ALT), aspartate transaminase (AST), total cholesterol (TC), low-density lipoprotein (LDL), high-density lipoprotein (HDL), and triglyceride (TG) were measured using the biochemistry analyzer (Hitachi, Tokyo, Japan). Reagents for glucose, ALT, AST, TC, LDL, HDL, and TG detection were from Wako Pure Chemical Corporation, Japan. The quality control materials were provided by Beckman Company (M507471 and M507473), and the calibrators were provided by Wako Pure Chemical Corporation, Japan.

### Genotyping

Genomic DNA was extracted from venous blood leukocytes using the EZ1 DNA Blood 350 μL kit (Qiagen) according to the manufacturer’s instructions for genotyping. Three SNPs, including rs1260326, rs780093, and rs780094 in *GCKR* from the NCBI database of SNP database (www.ncbi.nlm.nih.gov/SNP), were analyzed and genotyped by matrix-assisted laser desorption/ionization time-off light mass spectrometer in MassARRAY Analyzer 4 platforms (Sequenom, San Diego, CA). Probes and primers were determined with online Assay Design Suite version 2.0 software. The polymerase chain reaction was performed according to the instructions of the manufacturers. More detailed information about primers and polymerase chain reaction conditions is available upon request.

### Statistical analysis

Clinical data in subjects were presented as mean ± standard deviation. Independent samples t-test was adopted for the group comparison. Categorical data were calculated as a percentage. Each variable in this study satisfied the normality assumption distribution (SPSS statistical software version 26). Allelic and genotypic distributions and Hardy–Weinberg equilibrium were analyzed with the online software SHEsis (http://analysis.bio-x.cn/myAnalysis.php)^35^.

The association between each SNP with waist circumference in five genetic models (codominant, dominant, recessive, over-dominant, and log-additive models, respectively) was analyzed by “SNPassoc” R package^38^. These preliminary analyses identified relevant SNP genotypes (i.e., rs1260326) to lean NAFLD. The linear regression analysis was also conducted to verify the association of waist circumference with rs1260326 in lean NAFLD after the adjustment of gender and age (R script was shown in supplementary Table S2). Only those significant variables in a regression and genetic association analysis will be considered for the following mediation analysis. Mediation models conducted with mediation package (with the linear and generalized linear models) were established to check whether the waist circumstance can mediate the association between SNP and the odds of lean NAFLD by the mediation package in R software. P < 0.05 was considered statistically significant.

### Ethics approval and consent to participate

The study was approved by the Ethics Committee of the Shanghai University of Traditional Chinese Medicine. All participants provided informed written consent prior to the study.

## Supplementary Information

Supplementary Information 1.

Supplementary Information 2.

## Data Availability

All data can be obtained from the corresponding author’s request and public repository (https://pan.baidu.com/s/1HfMr8_DZFe7luh2PXVnUzw, code: e336).

## Abbreviations

ALT: Alanine aminotransferase
ANGPTL4: Angiopoietin-like protein 4
AST: Aspartate aminotransferase
BMI: Body mass index
Chr: Chromosome
CI: Confidence interval
DBP: Diastolic blood pressure
FDR: False discovery rate
FTO: Fat mass and obesity-related
GCKR: Glucokinase regulator
GWASs,: Genome-wide association studies
HCC: Hepatocellular carcinoma
HDL: High-density lipoprotein
HSD17B13: Hydroxysteroid 17-beta dehydrogenase 13
HWE: Hardy-Weinberg equilibrium
LDL: Low-density lipoprotein
NAFLD: Non-alcoholic fatty liver disease
NASH: Nonalcoholic steatohepatitis
OR: Odds ratio
PNPLA3: Patatin‐like phospholipase domain containing 3
SBP: Systolic blood pressure
SLC39A8: Solute carrier family 39 member 8
SNP: Single nucleotide polymorphisms
SUMO4: Small ubiquitin-like modifier 4
T2D: Type II diabetes
TC: Total cholesterol
TG: Triglyceride
TM6SF2: Transmembrane 6 superfamily member 2
WHR: Waist to hip ratio
