# Supplementary material for: Waist circumference mediates the association between rs1260326 in GCKR gene and the odds of lean NAFLD
Source: Sci Rep. 2023 Apr 20;13:6488. doi: 10.1038/s41598-023-33753-4 (PMC10119110; doi:10.1038/s41598-023-33753-4)
Supplement: Supplementary file 1 — Supplementary Information 1. [file 41598_2023_33753_MOESM1_ESM.docx]

**Supplementary Table 1.** Demographic data of lean NAFLD and lean non-NAFLD individuals

|  | **Lean NAFLD** | **Lean non-NAFLD** |
| --- | --- | --- |
|  | **N (%)** | **N (%)** |
| N | 106 | 216 |
| Current smoking |  |  |
| Yes | 3 | 10 |
| No | 103 | 206 |
| Alcohol consumption |  |  |
| Yes | 9 | 12 |
| No | 97 | 204 |
| Osteoporosis |  |  |
| Yes | 2 | 5 |
| No | 104 | 211 |
| Cerebral infarction |  |  |
| Yes | 3 | 0 |
| No | 103 | 216 |
