# Supplementary material for: Waist circumference mediates the association between rs1260326 in GCKR gene and the odds of lean NAFLD
Source: Sci Rep. 2023 Apr 20;13:6488. doi: 10.1038/s41598-023-33753-4 (PMC10119110; doi:10.1038/s41598-023-33753-4)
Supplement: Supplementary file 2 — Supplementary Information 2. [file 41598_2023_33753_MOESM2_ESM.docx]

Supplementary Table 2. R script

| R script for the regression analysis between waist circumference and rs1260326 adjusted by gender and age | rs <- dat_l$rs12603026  rs[which(rs == "TT")] <- "CC"  mod <- lm(waist circumference ~ rs + gender + AGE, data = dat_l_t)  summary(mod)  ci= exp(confint.default(mod))  or=exp(coef(mod))  ci  or |
| --- | --- |
